# Motivational interviewing for reducing rehospitalisation and improving patient activation among patients with heart failure or chronic obstructive pulmonary disease: a randomised controlled trial

**DOI:** 10.1136/bmjopen-2023-081931

**Published:** 2025-04-14

**Authors:** Monica Kaltenbrunner, Maria Flink, Carina Brandberg, Amanda Hellström, Mirjam Ekstedt

**Affiliations:** 1 Linnaeus University Faculty of Health and Life Sciences, Kalmar, Sweden; 2 University of Gävle Faculty of Health and Occupational Studies, Gavle, Sweden; 3 Neurobiology, Care Sciences and Society, Karolinska Institutet, Stockholm, Sweden; 4 Department of Social Work, Karolinska University Hospital, Stockholm, Sweden; 5 Capio S:t Gorans Hospital, Intensive Care Unit, Stockholm, Sweden; 6 LIME, Karolinska Institutet, Stockholm, Sweden

**Keywords:** Health Services, Health Services for the Aged, Nursing Care, Quality in health care, Aged

## Abstract

**AbstracBSTRACT:**

**Objectives:**

The aim is to evaluate the effects of a motivational interviewing-based intervention, Supporting Patient Activation in Transition to Home, on rehospitalisation and patient activation among patients with heart failure or chronic obstructive pulmonary disease.

**Design:**

A randomised, controlled, analysis-blinded trial was conducted.

**Setting:**

Participants were recruited from two hospitals in mid-Sweden and the intervention and interviews were conducted post-discharge.

**Participants:**

207 participants with heart failure or chronic obstructive pulmonary disease were recruited. Participants were randomised to receive five motivational interviewing sessions post-discharge (n=103) or a control group (n=104).

**Outcome measures:**

Rehospitalisation within 180 days post-discharge was retrieved, and patient activation was assessed using the Patient Activation Measure at baseline, 30, 90 and 180 days post-discharge. We used a generalised estimating equation to assess the difference in the secondary outcome, patient activation, between the intervention group and the control group during the 180-day follow-up.

**Results:**

No statistically significant differences between the groups were found for rehospitalisation (p=0.33 to 0.41) or patient activation over time (B=−1.67, –0.71 and −0.83 (95% CI −5.45 to 2.10, −4.06 to 2.64 and −4.28 to 2.62), respectively).

**Conclusion:**

Post-discharge motivational interviewing to decrease rehospitalisation or support patient activation does not seem beneficial for patients with heart failure or chronic obstructive pulmonary disease. The high disease burden may have limited patient participation in the intervention.

**Trial registration number:**

NCT02823795.

**STRENGTHS AND LIMITATIONS OF THIS STUDY**

- A strength of this study is the randomised controlled trial design with repeated measures during a 6-month follow-up, allowing for analysis of differences between groups over time.
- A key strength of the intervention is its flexibility, which was essential for participation due to the high disease burden and the advanced average age of the participants.
- The flexibility of the intervention limits the study’s validity.
- Another limitation is that the implementation of a double-blinded design was not feasible.

## Introduction

 Patients with chronic diseases like heart failure or chronic obstructive pulmonary disease are common in transitional care as they have a high risk of needing emergency care or being readmitted to hospitalisation.^1 2^ Transitional care, that is, patient transfers between healthcare providers, is associated with increased vulnerability to worsening health, medication-related harm and avoidable rehospitalisation.^1 3 4^ It can be challenging for patients to be involved and participate in transitional care due to comorbidities, poor health and decreased cognitive function.^5^ Patients and their carers may have difficulties understanding instructions in a discharge care plan.^6 7^ Therefore, patients often struggle after hospital discharge due to uncertainty regarding symptoms or treatment, a lack of information about self-management and follow-up appointments, and understanding and dealing with medication management.^8^,^10^

A wide variety of interventions has been tested to reduce rehospitalisation: telephone follow-up, discharge planning, medication reconciliation, patient education and complex interventions with several interacting components.^11^,^14^ Complex interventions with both pre- and post-discharge activities that support patient empowerment and capacity for self-management seem to be most effective for preventing rehospitalisation within 30 and 90 days post-discharge.^12 13^ This is in line with studies showing that patients with high levels of patient activation, that is, knowledge, skills and confidence in managing care independently at home, have improved health outcomes and reduced healthcare costs and care utilisation.^15^ A recent review showed that post-discharge interventions reduced hospitalisation for patients with heart failure or chronic obstructive pulmonary disease.^11^ However, the effectiveness of self-management interventions for patients with heart failure depends largely on a patient’s capacity to integrate the practices into daily life and to recognise disease-specific symptoms and signs.^16 17^

Motivation is key for patients to make behavioural changes, such as increased activation in self-management,^18^ meaning that introducing motivational interviewing in transitional care may be beneficial. Motivational interviewing is a person-centred method based on collaborative conversations and aims to strengthen a person’s motivation for change.^18^ A growing body of evidence shows that motivational interviewing is effective for behavioural change.^19^,^23^ For instance, Lundahl and Burke^22^ reported that motivational interviewing was effective for treating behavioural problems like substance use, reducing risky behaviours and increasing client engagement. Motivational interviewing seems effective regardless of age, gender or problem severity, and outcomes remain up to 1 year post-treatment. The greater the number of motivational interviewing sessions performed, the greater the behavioural change. One-to-one sessions seem more effective than group sessions.^22^

Few studies have tested motivational interviewing for reducing rehospitalisation among older people with severe conditions like heart failure or chronic obstructive pulmonary disease post-discharge. However, a pilot randomised controlled trial (RCT) of patients with heart failure showed modest but significant reductions in rehospitalisation,^24^ whereas a cluster RCT in primary care of patients with heart failure showed no difference in hospitalisation rates.^25^ An RCT of patients with chronic obstructive pulmonary disease showed significantly fewer rehospitalisations.^26^ The heterogeneity between the studies makes it impossible to draw firm conclusions, and more RCTs are needed to further elucidate the effects of motivational interviewing on rehospitalisation.^27^ The Supporting Patient Activation in Transition to Home (sPATH) was developed^28^ to test the effects of a motivational interviewing-based intervention on rehospitalisation and patient activation. The aim of the present study was to evaluate the effect of the sPATH intervention on rehospitalisation and patient activation among patients with heart failure or chronic obstructive pulmonary disease.

## Method

### Study design and setting

This randomised, controlled, analysis-blinded trial^28^ was conducted at four healthcare departments at two hospitals in mid-Sweden: the emergency department and the lung department at Karolinska University Hospital and the emergency department and the cardiology department at Capio S:t Göran Hospital (online supplemental file 1).^28^ The study followed the Consolidated Standards of Reporting Trials guidelines for reporting randomised trials.^29^

### Study population, recruitment and randomisation

**Figure 1:**
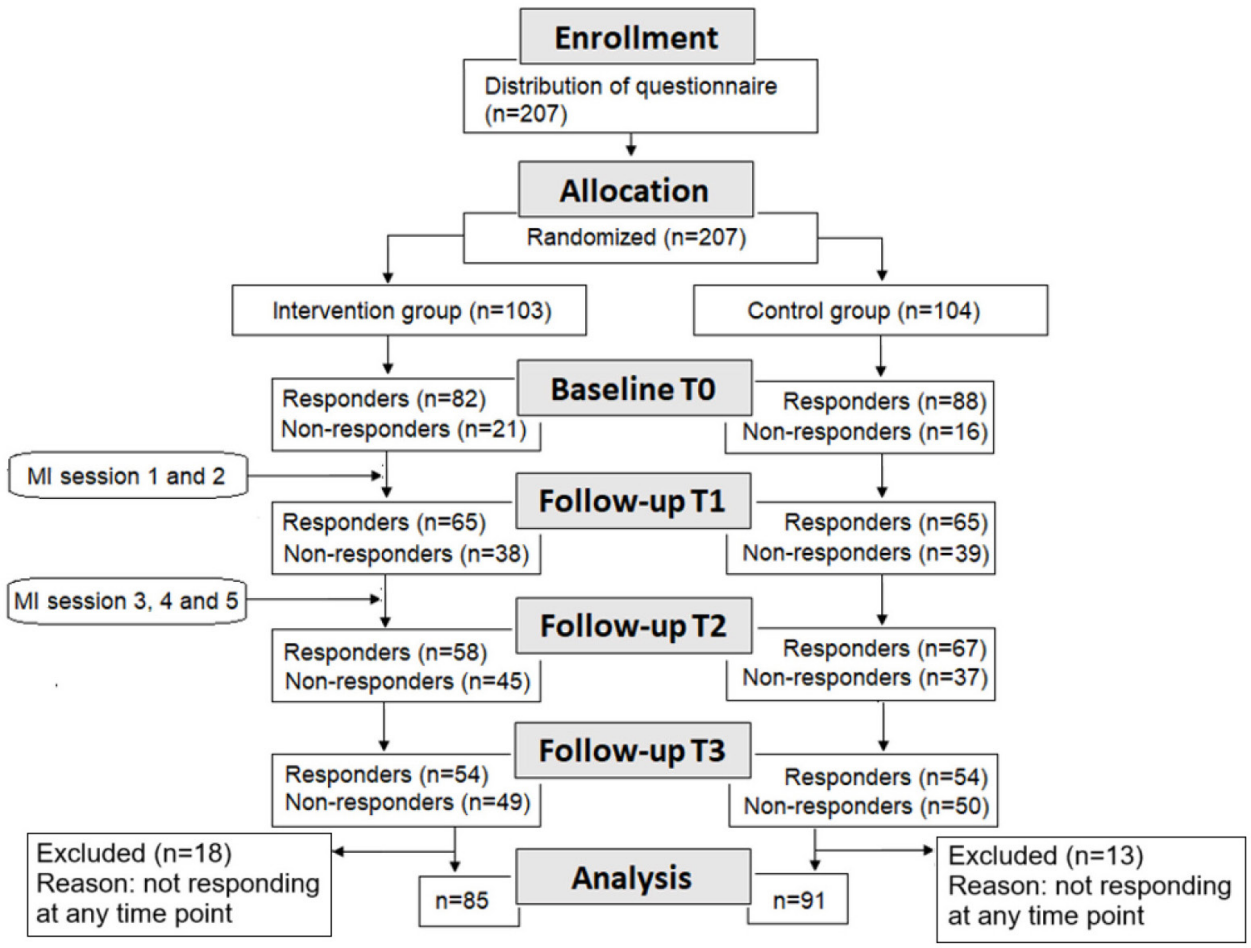
Flow diagram of the study. The questionnaire was distributed at baseline (**T0**) and T1–T3. Analyses of baseline characteristics, clinical data, self-rated health and rehospitalisation included 207 patients, and analyses of Patient Activation Measure included 176 patients in total. MI, motivational interviewing.

Recruitment started in August 2016 and was terminated in May 2018.^28^ Eligibility criteria were patients admitted to hospitals, who were diagnosed with congestive heart failure or chronic obstructive pulmonary disease, being aged at least 18 years, and living in ordinary housing. Exclusion criteria were having a statement of ‘do not resuscitate’ in their medical record, needing an interpreter and/or having a diagnosis of dementia or cognitive impairment. Information on patients’ diagnoses was retrieved from their medical records. Eligible patients were identified by nurses or physicians at the departments; recruitment was conducted by two researchers (MF and CB). Eligible patients received both written and verbal information about the study and confidentiality from the researchers. Each participant gave consent to participate on a written form. The participants received the baseline questionnaire before randomisation. An 8-block randomisation was conducted, dividing patients into four intervention subgroups and four control subgroups. A randomisation list was developed by an independent statistician familiar with randomisations. The study was an open-label study, as the type of treatment could not be blinded to those involved. See figure 1 for responders and non-responders throughout the study as well as activities in the interventions. Non-responders are participants who do not respond to the questionnaire for any reason.

### sPATH intervention

The intervention was developed based on self-determination theory,^30^ with the aim to enhance skills and confidence in self-management post-discharge and to motivate patients to take a more active role or remain active in self-management. The intervention encompassed five post-discharge motivational interviewing sessions either by telephone or face-to-face. The sessions were led by three coaches who were medical social workers, with education and experience in motivational interviewing and knowledge of the intervention. The coaches received monthly mentoring from a trained coach, a member of the Motivational Interviewing Network of Trainers, during the intervention.^31^ Four self-management activity areas were discussed at the motivational interviewing sessions, based on evidence from successful care transition interventions.^32^ At the first session, the focus was on detecting acute problems and scheduling the other four sessions. The subsequent sessions were centred around the following main areas, adapted to the patient’s choice: (1) medication management, (2) adherence to care plan/follow-up visits connected to the discharge plan, (3) recognising and handling symptoms and signs worsening the condition, (4) contacting healthcare providers and dealing with relationships and meetings with them.^28^ Each patient could choose which area they wanted to discuss during a session or suggest another topic important to them, in order to be active in post-discharge self-management. The intervention was standardised, although it allowed adjustment based on a patient’s needs for knowledge, competence and skills in managing self-care.^30 33^

Inclusion and randomisation were conducted consecutively, meaning that the intervention started on different dates for the participants. The five sessions of motivational interviewing were conducted during a period of 1.5–2 months. The first was held by telephone within a few days after discharge from the hospital.^8^ The second session was held about 1 week after discharge, and the remaining sessions were held at 1-week intervals. Patients in the control group received care as usual; that is, at discharge, they were given a medication list and a discharge letter containing information on the hospital care episode, plans for follow-up and contact information on whom to contact with any questions.

### Data collection and outcomes

Questionnaires were filled out at four time points: baseline (T0) and 30 (T1), 90 (T2) and 180 (T3) days after baseline, respectively. At T0, before randomisation, MF and CB collected baseline data. Due to patient fatigue and/or time constraints related to discharge, some patients were given the option to complete the questionnaire at home. After handing out the questionnaires, MF and CB sent a notification via a secure link to the coaches to inform them that the intervention could start. To assess each patient’s perception of the care transition, a separate questionnaire was distributed in connection to discharge from the hospital. Patients were asked to respond within 1 week and return the questionnaire in a postage-paid return envelope. Two reminders were sent to non-responders. Before sending the final reminder, at least one attempt was made to contact each patient by telephone or text message.

### Outcomes

The primary outcome measure was ‘rehospitalisation’ and included three variables: (1) the ‘number of nights at home’ between discharge from index admission to first rehospitalisation, (2) the ‘number of hospital admissions’ and (3) the ‘total number of hospital nights’ as an inpatient between discharge from index admission and 180 days later. These data were retrieved from the Stockholm region’s register for healthcare encounters.

The secondary outcome measure, patient activation, was assessed at baseline, 30 days, 90 days and 180 days, using the 13-item Patient Activation Measure (PAM).^33^ The instrument assesses a patient’s self-reported skills, knowledge and confidence for self-management of health and healthcare and has been used among a wide range of patients.^34^,^36^ Responses are given on a 4-point Likert scale from ‘strongly disagree’ to ‘strongly agree’, where higher scores indicate higher levels of activation. The Swedish version of the PAM has been validated,^37^ with a Cronbach’s alpha of 0.81, and is considered reliable—though further development to increase validity is recommended.

In the study protocol, medication adherence was included as a secondary outcome. However, due to licensing issues with the instrument we intended to use, we decided not to include this outcome measure. Since the issue arose after data collection, no alternative instrument could be used instead.

### Statistical analysis

IBM SPSS V.27 was used to analyse data. Normal distribution of data was checked using histograms. Differences between the intervention and control groups were examined with independent samples t-tests for continuous variables, and Pearson’s χ^2^ tests for categorical variables. To assess differences in the primary outcome, ‘rehospitalisation’, between intervention and control groups, as well as the longitudinal effect of the sPATH intervention on ‘rehospitalisation’, the Mann-Whitney U test was used, as the variable was not normally distributed.

To assess the difference in the secondary outcome, PAM score, between the intervention group and the control group during the 180-day follow-up, a generalised estimating equation (GEE) was used.^38^ We generated two models with PAM score as the dependent variable. The variables tested in the first model were time, group and the interaction time×group. In the second model, we included the same variables as in model 1, and the independent variables were age, female gender, having social support and the Charlson Comorbidity Index (CCI). GEE adjusts for within-person correlations in responses. The working correlation structure was exchangeable.

For the PAM items, missing values were handled using multiple imputation. This method generated five datasets with imputed data values in addition to the original dataset.^38^ In the analysis, results are received from each dataset, and a pooled result is based on all five datasets. To study the sensitivity, all analyses were also conducted using the original, not imputed, data. P values<0.05 were considered statistically significant.

Follow-up data in the PAM were missing from some participants. An analysis of these non-responders (n=31 participants) (figure 1) showed no statistically significant differences between them and responders as regards age (p=0.909), gender (p=0.945) or CCI (p=0.779).

### Patient and public involvement

None.

## Results

The aim was to recruit 121 patients for each group, to ensure adequate statistical power.^28^ However, recruiting was terminated when a total of 207 participants was included, to finish data collection within a reasonable time. The data collection was prolonged as a result of challenges in recruiting patients due to their severe conditions and fatigue.^7 39^ In total, 103 patients were randomised to the intervention group and 104 to the control group (figure 1).

A total of 37 patients terminated their participation during the trial. Of these, 26 patients left the study due to death, illness, fatigue, confusion, announced drop-out or an unknown reason. In total, 31 patients (of whom 18 were in the intervention group) were not included in the analysis as they did not respond at any of the four data collection points, baseline (T0), 30 days (T1), 90 days (T2) and 180 days (T3) post-discharge.

### Baseline characteristics

Baseline characteristics for participants in the intervention group and the control group are reported in table 1. A statistically significant difference between the intervention group and control group was found for CCI at baseline (p=0.049) as CI not included zero (0.003 to 1.15). This shows that CCI was higher in the intervention group than in the control group and reflects that the intervention group had a higher average disease burden. Baseline data are missing for 37 participants (intervention group n=21, control group n=16).

**Table 1:** Baseline characteristics, clinical data and self-rated health, internal consistency, and test of differences between intervention and control group (p value)

	Intervention group (n=103)	Control group (n=104)	P value
Men, n (%)	58 (56)	51 (49)	0.364*
Age, n	103	104	0.500†
Mean (SD)	75.3 (10.1)	74.3 (10.8)	95% CI −1.89 to 3.86
Median (Q_1_–Q_3_)	77 (69–82)	75 (68–83)	
Having social support, n (%)	75 (73)	74 (71)	0.358*
Income (SEK), n (%)			0.169*
<10 000	4 (4)	9 (9)	
10 000–20 000	32 (31)	43 (41)	
20 000–50 000	34 (33)	24 (23)	
>50 000	8 (8)	8 (8)	
Education, n (%)			0.553*
< 9 years	5 (5)	6 (6)	
Primary school (9 years)	21 (20)	17 (16)	
Secondary school	26 (25)	39 (38)	
University	28 (27)	23 (22)	
Country of birth, n (%)			0.901*
Sweden	70 (68)	71	
Nordic country other than Sweden	6 (6)	5 (5)	
Outside the Nordic countries	5 (5)	8 (8)	
CCI, n	94	91	**0.049** †
Mean (SD)	6.2 (2.0)	5.7 (2.0)	95% CI 0.003 to 1.15
Median (Q_1_–Q_3_)	6 (5–7)	6 (5–7)	
PHQ-9 (α=0.82), n	73	80	0.412*
Mean (SD)	8.5 (5.2)	9.1 (6.5)	
Median (Q_1_–Q_3_)	8 (5–12)	8 (4–13)	
EQ (α=0.76), n	78	86	0.744*
Mobility			
Median (Q_1_–Q_3_)	3 (2–4)	3 (2–4)	
Self-care	78	86	0.392*
Median (Q_1_–Q_3_)	1 (1–2)	1 (1–2)	
Usual activity	77	87	0.754*
Median (Q_1_–Q_3_)	3 (2–4)	2 (2–4)	
Pain/discomfort	78	86	0.331*
Median (Q_1_–Q_3_)	3 (2–3)	3 (2–3)	
Anxiety/depression	77	87	0.806*
Median (Q_1_–Q_3_)	2 (1–3)	2 (1–3)	

If the numbers in some columns do not add up to 104 or 103, it is because some of the participants did not respond. Significant p values are highlighted in bold.

*χ 2Chis-square test for independence.

† Independent samples t-test.

CCI, age-adjusted Charlson Comorbidity Index; EQ EQ-5D-5L, European Quality of Life 5 Dimensions 5 Levels; PAM, Patient Activation Measure-13-S; PHQ-9, Patient Health Questionnaire; Q, quartile; SEK, Swedish krona; α, Cronbach’s alpha.

Almost all patients randomised to the intervention underwent all five motivational interviewing sessions. Most of the patients chose to have the sessions via telephone instead of face-to-face due to fatigue and severe disease.

### Intervention effects on rehospitalisation

There were no statistically significant differences between the intervention group and the control group in any of the three measures regarding rehospitalisation (p values from 0.334 to 0.408) (table 2).

**Table 2:** Mean and median differences between the intervention group and control group in rehospitalisation during the 180-day follow-up after discharge

N=103/104	Number of nights at home before first rehospitalisation			Number of rehospitalisations			Number of hospital nights		
	IG	CG	P value	IG	CG	P value	IG	CG	P value
Mean (SD)	47.4 (45.6)	51.2 (43.4)	0.408	2.8 (3.2)	3.3 (4.0)	0.334	28.8 (28.8)	32.1 (31.3)	0.390
Median (Q_1_–Q_3_)	35 (10–72)	38 (18–80)		2 (0–4)	2 (1–4)		20 (6–45)	21 (7–51)	

CG, control group; IG, intervention group; Q, quartile.

The control group had more nights at home between T0 and first rehospitalisation (mean 51.2, SD 43) than the intervention group (mean 47.4, SD 46). The number of rehospitalisations and the number of hospital nights were slightly higher in the control group (mean 3.3, SD 4 and mean 32.1, SD 31, respectively) than in the intervention group (mean 2.8, SD 3 and mean 28.8, SD 29, respectively).

### Intervention effect on patient activation

The multivariate analysis for model 1 showed no support for there being differences between the intervention group and the control group when testing the interaction time×group, meaning that no differences in the PAM score were found between the groups over time (p values from 0.381 to 0.678) (table 3). The multivariate analysis was also conducted for model 2, including the control variables age, gender, social support and CCI, showing similar results as in model 1, and none of the control variables were significant (p values from 0.054 to 0.927).

**Table 3:** Results of GEE, model 1 and model 2, showing the intervention effect on PAM score at 30, 90 and 180 days post-discharge

Model 1				Model 2		
Parameter estimates						
Parameter	B	95% CI lower and upper	P value	B	95% CI lower and upper	P value
Intercept	55.031			59.206		
T1	1.734	−0.859 to 4.327	0.188	1.978	−0.955 to 4.912	0.185
T2	2.321	−0.390 to 5.014	0.093	2.321	−0.820 to 5.462	0.145
T3	3.061	0.226 to 5.895	**0.035**	3.023	0.352 to 5.695	**0.027**
Group (control)	1.8115	−1.611 to 5.242	0.297	1.183	−2.197 to 4.563	0.493
T1×group (control)	−1.673	−5.445 to 2.099	0.381	−1.725	−5.824 to 2.374	0.407
T2×group (control)	−0.710	−4.063 to 2.643	0.678	−0.914	−5.056 to 3.228	0.663
T3×group (control)	−0.832	−4.281 to 2.617	0.634	−0.433	−4.053 to 3.187	0.814
Gender				−0.722	−4.221 to 2.776	0.681
Relative				5.044	−0.277 to 10.366	0.063
Age				0.011	−0.237 to 0.258	0.927
CCI				−1.526	−3.084 to 0.032	0.054

Dependent variable: PAM. T1, 30 days post-discharge; T2, 90 days post-discharge; T3, 180 days post-discharge.

The variables T0 and group (intervention) were used as references in the analyses.

CCI, Charlson Comorbidity Index; GEE, generalised estimating equation; MI, motivational interviewing; PAM, Patient Activation Measure.

A statistically significant association was found between PAM score and time (p=0.035) (table 3), meaning that PAM scores increased between T0 and T3 for the total sample. For the intervention group, the mean increased from 54.7 (SD 12) at T0 to 59.0 (SD 11) at T3 (table 4). For the control group, the mean increased from 56.6 (SD 11) at T0 to 58.9 (SD 14) at T3. The multivariate analysis was also performed using original, not imputed data. These analyses showed similar results as those based on imputed data.

**Table 4:** PAM score, mean and median over time for the intervention and control groups

PAM score	T0	T1	T2	T3
Intervention group				
Mean (SD)	54.7 (11.8)	57.4 (12.1)	57.4 (12.7)	59.0 (10.5)
Median (Q_1_–Q_3_)	51 (47–63)	53 (50–68)	58 (49–66)	59 (51–68)
Control group				
Mean (SD)	56.6 (11.0)	55.9 (12.1)	56.9 (13.6)	58.9 (13.7)
Median (Q_1_–Q_3_)	56 (49–63)	53 (48–63)	56 (47–63)	58 (49–66)

Descriptive data showing PAM scores for the intervention group and control group at baseline, 30, 90, and 180 days. Internal consistency of the PAM.

PAM, Patient Activation Measure; Q, quartile; α, Cronbach’s alpha.

## Discussion

This study reports the results of an RCT that investigated the effects of sPATH, a motivational interviewing intervention, on rehospitalisation and patient activation in patients with heart failure or chronic obstructive pulmonary disease. We did not find any statistically significant differences between the intervention and control groups in rehospitalisation or patient activation. Our results thus add to those of previous studies, showing that motivational interviewing leads to no reduction of rehospitalisation,^40^ though they contradict other studies in which motivational interviewing interventions reduced rehospitalisation^24 26^ and improved patient activation.^41 42^

A result that deserves to be highlighted is that PAM scores increased over time for both groups, although the participants had high disease burdens and mean age. The within-group increase in PAM scores was greater in the intervention group, who recovered to the same level as the control group at 180 days despite suffering from a higher illness burden at baseline. Although motivational interviewing has the potential to be fruitful, it needs to be considered that it can be challenging to involve participants with a high disease burden (CCI>6) and high mean age (>75 years), as these factors may affect willingness for participation.^39^ In our study, the mean CCI was statistically significantly higher in the intervention group than in the control group at baseline, which may have limited those patients’ ability to assimilate the intervention.^39^ A longitudinal qualitative analysis of the motivational interviewing sessions based on a convenience sample of participants from the intervention group^43^ showed that they perceived severe fatigue, anxiety and stress related to the disease burden in the first 2–4 weeks after discharge.^43^ Both the disease burden and the perceived health in our sample need to be considered in relation to the lack of effect of the motivational interviewing intervention with respect to rehospitalisation. A study of 56 patients with heart failure and comorbidities showed that perceived ill health, but not laboratory measures, was associated with rehospitalisation,^44^ indicating that patients’ perceptions of their health influenced health-seeking behaviour. Further, a study of patients (mean age 61 years) with chronic obstructive pulmonary disease showed that rehospitalisation was two times as common in a control group compared with an intervention group receiving a motivational interviewing intervention.^45^ However, the significant difference disappeared when adjusting for hospitalisations within 12 months before the trial.^45^ This indicates that a patient’s medical history is also an important factor affecting re-hospitalisation.

As regards our reasoning that patient age might influence our results, another study of older patients (mean age 85 years) with heart failure found no differences between intervention and control groups in hospitalisation rates after a motivational interviewing intervention.^25^ Studies involving younger patients reported other results—for instance, in two RCTs based on samples with lower mean age (68 and 60 years, respectively) than ours, the intervention groups had lower rehospitalisation rates than the controls.^24 26^ A systematic review on medication adherence showed that motivational interviewing interventions were more effective among older participants. The median age in that study was 59 years,^20^ which can be considered young compared with our sample.

For patients with severe disease and high age, rehospitalisation may be the only option for feeling safe and secure when health deteriorates. The reasons for not feeling safe and secure can be several, and various shortcomings in the healthcare system may influence this. For instance, deficiencies in connection to discharge have been reported, where patients received incomplete information about which care provider to seek if symptoms worsened or complications occurred.^5 46^ Other shortcomings concern that patients, after discharge from the hospital, struggle with understanding and managing symptoms, treatment and follow-up appointments.^8^,^10^ Several studies underline that patients with severe disease do not feel safe and secure in that they will receive healthcare at home when needed.^6^,^9^ Therefore, they seek help at an emergency department when they need healthcare urgently.^10^

The results call for reflection on the challenges of designing an intervention for older adults who are managing severe illness at home. Our intervention encompassed five one-to-one motivational interviewing sessions, which are more effective than group sessions, according to Lundahl and Burke.^22^ As it has been reported that more sessions increase behavioural change,^22^ future studies could explore if increasing the number of sessions might be helpful for reducing rehospitalisation. Almost all participants chose to have the sessions via telephone instead of face-to-face—an approach that is less effective, according to a systematic review and meta-analysis.^23^ Recent studies of follow-up telephone calls aiming to reduce rehospitalisation after hospital stays, using methods other than motivational interviewing, have shown inconsistent results.^47 48^

Although the design of our intervention was complex^49^ and supported the patients’ capacity for self-management, in line with previously suggested best practices,^13^ no differences were seen between the intervention and control groups. The intervention was stand-alone, without including patients’ healthcare providers or any pre-discharge activities. Thus, in line with previous findings, our study suggests that both pre- and post-discharge activities are needed to prevent rehospitalisation through care transition interventions.^12 13^

### Strengths and limitations

The study has both strengths and limitations. A limitation is that the intervention was not developed in collaboration with patients with heart failure or chronic obstructive pulmonary disease. Instead, it was developed based on self-determination theory and the principles of autonomy support,^30^ an adaptation proven to be successful in several studies,^32^ care transition literature^13 47 48^ and pre-studies.^8 50 51^ Therefore, no feasibility study was conducted to assess the patient acceptability of the intervention. One important limitation is that we were not able to report the primary outcome rehospitalisation rates, as stated in the study protocol,^28^ because we could only receive individual-level register data from the Stockholm region’s register for healthcare encounters. Instead, we have provided information on the number of rehospitalisations. Another limitation concerns the drop-out rates, which could have influenced the overall results. Specifically, 37 individuals did not complete the study due to serious issues such as death, illness or confusion, indicating the high disease burden among this population. Moreover, one limitation concerns session execution. The coaches providing motivational interviewing sessions were hospital social workers, trained in motivational interviewing, but without medical education. This might have resulted in the sessions focusing on more general aspects of self-management rather than aspects measured by the PAM, such as symptom and medication management. As we did not review the coaches’ fidelity to the intervention, we have no data on what the sessions focused on. However, motivational interviewing is person-centred at its core and the intervention therefore included flexibility for the coaches to focus the sessions on what each patient perceived to be most important. The coaches received ongoing supervision in both motivational interviewing and the study’s purpose throughout the trial. The coaches were not familiar with the patients beforehand and there were no plans for long-term contact beyond the intervention, which possibly decreased engagement among the patients. Including older adults with severe diseases in interventions is challenging^41 52^—they are often excluded from studies for that reason. The flexibility of our intervention regarding meeting patient needs was both a strength and a limitation. In the motivational interviewing sessions, each patient was to some extent a co-creator of the intervention as their preferences steered the content with the overarching aim of decreasing rehospitalisation through increased patient activation. The flexibility also included having the sessions via telephone or face-to-face. This flexibility was necessary to get patients to participate in the study, but is a limitation to the study’s validity. By having a ‘pragmatic’ attitude to trial design—making the interventions as similar to what they would be in routine healthcare as possible—we sought to make the results more applicable to the real-world context.^53^ This implies that judgement must be used to interpret the degree to which these results apply to any other given situation, patient or healthcare setting. As intervention studies in a population with severe disease and recently hospitalised patients are rare, this study has—despite its limitations—led to new understandings about the challenges and limited success that motivational interviewing might have for older adults with heart failure or chronic obstructive pulmonary disease. Furthermore, the trial was underpowered due to recruitment difficulties and early termination, meaning that conclusions must be interpreted carefully. Finally, the study outlined in the trial registry^28^ is a comprehensive project with multiple outcome measures, some of which have already been reported in other publications. Although this study uses data collected from the trial, it serves a distinct purpose and, consequently, does not fully address all the objectives stated in the protocol. For example, not all outcome measures were included in this study.

## Conclusions

Motivational interviewing to decrease rehospitalisation or support patient activation does not seem to benefit patients with heart failure or chronic obstructive pulmonary disease post-discharge. The high disease burden may have limited patients’ participation in the intervention. Future studies are needed to investigate if older patients with severe illness would benefit more from receiving post-acute care, such as skilled home healthcare and intermediate care after discharge, rather than self-care support.

## Supplementary material

10.1136/bmjopen-2023-081931 online supplemental file 1

## Data Availability

All data relevant to the study are included in the article. No data are available.
