# Supplementary material for: Motivational interviewing for reducing rehospitalisation and improving patient activation among patients with heart failure or chronic obstructive pulmonary disease: a randomised controlled trial
Source: BMJ Open. 2025 Apr 14;15(4):e081931. doi: 10.1136/bmjopen-2023-081931 (PMC11997831; doi:10.1136/bmjopen-2023-081931)
Supplement: Supplementary file 2 [file bmjopen-2023-081931.reviewer_comments.pdf]

PEER REVIEW HISTORY

BMJ Open publishes all reviews undertaken for accepted manuscripts. Reviewers are asked to complete a checklist review form and are provided with free text boxes to elaborate on their assessment. These free text comments are reproduced below.

ARTICLE DETAILS

Title (Provisional)

Motivational interviewing for reducing rehospitalization and improving patient activation among patients with heart failure or chronic obstructive pulmonary disease: A randomized controlled trial

Authors

Kaltenbrunner, Monica; Flink, Maria; Brandberg, Carina; Hellström, Amanda; Ekstedt, Mirjam

---

VERSION 1 - REVIEW

Reviewer 1

Name Wu, Yongjian

Affiliation National Clinical Research Center of Cardiovascular Diseases, State Key Laboratory of Cardiovascular Disease, Fuwai Hospital, National Center for Cardiovascular Diseases, Chinese Academy of Medical Sciences and Peking Union Medical College, Beijing, People's Republic of China

Date 11-Jan-2024

COI None

Thank you for giving me the opportunity to review this manuscript.
In this study, the authors tried to clarify the impact of a motivational interviewing intervention on the prognosis in patients with heart failure or chronic obstructive pulmonary disease through a randomized controlled study. The design and analysis plan of the study are well articulated; however, before accepting this paper, the author should address the following concerns:

1. In the introduction, the author spent a considerable amount of text on background information. This section should be concise and to the point.

2. In the methods section, the author mentioned that the sPATH intervention was conducted by three coaches, which introduces an issue of heterogeneity. Are there quality control measures and criteria in place to ensure consistency among the three coaches in the implementation of the intervention?

3. In the methods section, what does 'MI sessions' refer to? Please provide the full name when

using an acronym for the first time

4. In the methods section, the primary outcome was identified as the ‘rehospitalization rate’. In my understanding, the rehospitalization rate should be calculated as the number of re-hospitalized individuals divided by the total number of individuals in that group. However, the author described that the primary outcome included three components: 'number of nights at home', 'number of hospital admissions', and 'total number of hospital nights', all of which were numerical variables rather than proportions. This seems to deviate from the defined primary outcome. I am unclear why the author chooses these alternative numerical variables instead of the actual rehospitalization rate. Additionally, I also did not find these three components in the protocol published by the author. Is it because the primary endpoint was defined elsewhere that I have not discovered? Furthermore, is there any literature support for using these three components as the primary outcome to evaluate the effectiveness of the intervention?

5. In the study protocol, the author mentioned several secondary outcomes, such as depression, health-related quality of life, etc. However, I seem to be unable to find the results for these outcomes in the research paper.

6. In the methods section, the author proposed using the Mann-Whitney U test to examine the 'rehospitalization rate'. However, in the title of Table 2, the author mentioned using the t-test to compare differences between the two groups. Please explain the reason for this inconsistency

7. In the methods section, the author mentioned using two models to evaluate the relationship between the intervention and PAM. However, in Table 3, I only see the results for one model. Is this result from Model 1 or Model 2? I believe the author should present the results for both models.

8. In the last paragraph of the methods section, the author described the differences between non-responders and responders on PAM. I suggests the author provide an appendix table to clearly show the differences between these two groups, rather than merely stating them. And, this content should be moved to the results section.

9. In the methods section, the author noted that 37 individuals did not complete the study, with 26 of them dropping out due to death, illness, fatigue, or confusion. In Table 2, the author has compared the primary outcome for all enrolled patients (intervention/control group: n=103/104). How has the author considered the impact of those who did not complete the study on the primary outcome? For instance, the number of re-hospitalizations and length of stay in hospital of the patient who died within 180 days would be competed.

10. The author should improve the linguistic quality and rigor of the manuscript writing. For example, there is a confusing sentence: 'The number of rehospitalizations and hospital nights were slightly higher in the control group (mean 3.3, SD 4.0 respectively mean 32.1, SD 31.3) than in the intervention group (mean 2.8, SD 3.2 respectively (mean 28.8, SD 28.8)'. Additionally, there are some issues with punctuation and capitalization. Please revise the entire manuscript for these issues.

**Reviewer**	**2**
**Name**	**Spencer , Lissa M**
**Affiliation**	**Royal Prince Alfred Hospital**
**Date**	**17-Mar-2024**
**COI**	**No competing interests**

Thank you for your hard work in completing and writing up this study. Motivation is an important part of treatment adherence, especially if it could change readmissions. I have made some comments below.

Abstract:

• Line 29: Spelling mistake 'Objectives'

Introduction

• Line 86: define 'Transitional care'. You have not mentioned this in the Abstract. I don't think you should start the Introduction with Transitional care - perhaps you should start with paragraph two.

• Line 140: you have stated that 'The aim of the present study was to evaluate the effect of the sPATH intervention on rehospitalization rates and patient activation among patients with heart failure or chronic obstructive pulmonary disease'. However, in the Abstract, you have stated your aims as: 'The aim is to evaluate the effects of a motivational interviewing-based intervention, on rehospitalization and patient activation among patients with congestive heart failure or chronic obstructive pulmonary disease'. The aims should be the same and you need to mention sPATH in the Abstract.

Methods:

• Line 153: 'Eligibility criteria were that the patients should have been diagnosed with heart failure or chronic obstructive pulmonary disease…' Rewrite as: 'Eligibility criteria were patients who had been diagnosed with heart failure or chronic obstructive pulmonary disease…'

• You need to add how the diagnosis of COPD or HF was made, based on spirometry or echocardiogram results?

• Line 162 to 175: This is all Results and needs to go in the Results section.

• Line 166: 'a larger block would have balanced less well' This sentence does not really make sense. If you feel this is a limitation – keep limitations for the Discussion section.

• Line 209: You are getting results and methods confused. You need to list the outcomes collected and not comment on the number of Qs completed – this would go in the results.

• Line 223: Change this heading from 'Baseline Characteristics' to 'Outcomes' and list all outcomes here – make a new paragraph for each outcome. Baseline characteristics should not be mentioned until the first paragraph of the Results section.

• Primary outcome should be listed first, then secondary outcomes and then all the other questionnaires.

Results:

• Start the results section with: 'Baseline characteristics for participants in the IG and the CG are reported in Table 1'. Do not write it out – if it is in Tabel 1, that is sufficient. Then go straight to your important between group results.

• Table 1 needs a column showing the between group differences plus 95%CI.

• Line 317: a capital T for Table 2 and Table 3.

• In the Tables, you could abbreviate intervention group to IG and control group to CG.

• When presenting results – make all SD results into whole numbers.

• In Figure 1: what do the authors mean by responders and non-responders?

• Figure 2: more descriptive to have SD marked on the graph.

• Do the authors think that more sessions than five in the intervention group, would have improved results would have been different?

Discussion:

• Well done and limitations have answered some of my questions

Thank you again for your hard work.

VERSION 1 - AUTHOR RESPONSE

Reviewer: 1

Comments	Response	Page, line
1.In the introduction, the author spent a considerable amount of text on background information. This section should be concise and to the point.	Thank you for the suggestion to reduce the background information. We have followed your advice.	The introduction section,<br>p 4-5.
2.In the methods section, the author mentioned that the sPATH intervention was conducted by three coaches, which introduces an issue of heterogeneity. Are there	We agree that the lack of control measures on the coaches' fidelity to the intervention is a limitation. We have	P 15<br>Line 403-408

quality control measures and criteria in place to ensure consistency among the three coaches in the implementation of the intervention?	added a statement in the limitation section to address this issue.	
3.In the methods section, what does 'MI sessions' refer to? Please provide the full name when using an acronym for the first time	Thank you for noticing this. We have changed MI sessions to motivational interviewing sessions.	-
4.In the methods section, the primary outcome was identified as the ‘rehospitalization rate’. In my understanding, the rehospitalization rate should be calculated as the number of re-hospitalized individuals divided by the total number of individuals in that group. However, the author described that the primary outcome included three components: 'number of nights at home', 'number of hospital admissions', and 'total number of hospital nights', all of which were numerical variables rather than proportions. This seems to deviate from the defined primary outcome. I am unclear why the author chooses these alternative numerical variables instead of the actual rehospitalization rate.<br><br>Additionally, I also did not find these three components in the protocol published by the author. Is it because the primary endpoint was defined elsewhere that I have not discovered?<br><br>Furthermore, is there any literature support for using these three components as the primary outcome to evaluate the effectiveness of the intervention?	Thank you for highlighting this important issue. Unfortunately, although we have received the register data from Stockholm region’s Register for Healthcare Encounters, it is on an individual level. This limits us when choosing how to report data. As a result, we are unable to present rehospitalization rates at the organizational level as typically done.<br><br>However, we consider that these three components beneficially capture a thorough picture of rehospitalization.<br><br>The number of days spent in the hospital is a relevant metric, as are the days spent at home between the initial event and rehospitalization, which is also an important quality measure (considering patients may have been discharged to another clinic).<br><br>We have added some sentences concerning this in the limitation section to address this issue.	P 14<br>Line 392-399
5.In the study protocol, the author mentioned several secondary outcomes, such as depression, health-related quality of life, etc. However, I seem to be unable to find the results for these outcomes in the research paper.	Thank you for bringing this up. As you noted, the project we describe in the study protocol is extensive and includes repeated measures with both primary and secondary outcome variables. Since it was not feasible to include all variables in a single article, we chose to publish the primary outcomes and possible	-

	confounding factors in this article. A second article with secondary outcomes will be published in a forthcoming manuscript.	
6.In the methods section, the author proposed using the Mann-Whitney U test to examine the 'rehospitalization rate'. However, in the title of Table 1, the author mentioned using the t-test to compare differences between the two groups. Please explain the reason for this inconsistency	Thank you for highlighting this. We have now clarified the text in Table 1, stating which test is used for each variable.	Page 10, Table 1
7.In the methods section, the author mentioned using two models to evaluate the relationship between the intervention and PAM. However, in Table 3, I only see the results for one model. Is this result from Model 1 or Model 2? I believe the author should present the results for both models.	Thank you for addressing this. We have now added both models in Table 3.	Page 12, Table 3
8.In the last paragraph of the methods section, the author described the differences between non-responders and responders on PAM. I suggests the author provide an appendix table to clearly show the differences between these two groups, rather than merely stating them. And, this content should be moved to the results section.	Thank you for highlighting this uncertainty.<br>We only have data on PAM from those who responded. We don't have data for those who have not responded. To clarify this misunderstanding, we have revised the phrasing to make it clearer what we mean when using the words "responders" and "non-responders."	P 8<br>Line 249-250
9.In the methods section, the author noted that 37 individuals did not complete the study, with 26 of them dropping out due to death, illness, fatigue, or confusion.<br><br>In Table 2, the author has compared the primary outcome for all enrolled patients (intervention/control group: n=103/104). How has the author considered the impact of those who did not complete the study on the primary outcome? For instance, the number of re-hospitalizations and length of stay in hospital of the	Thank you for your insightful question. We acknowledge that the dropout rate, particularly among those who encountered serious issues such as death, illness, or confusion, could influence the overall results. We added a sentence about this as a limitation.	P 14<br>Line 396-399

patient who died within 180 days would be competed.		
10.The author should improve the linguistic quality and rigor of the manuscript writing. For example, there is a confusing sentence: 'The number of rehospitalizations and hospital nights were slightly higher in the control group (mean 3.3, SD 4.0 respectively mean 32.1, SD 31.3) than in the intervention group (mean 2.8, SD 3.2 respectively (mean 28.8, SD 28.8)'. Additionally, there are some issues with punctuation and capitalization. Please revise the entire manuscript for these issues.	Thank you for bringing this to our attention. We have rewritten the sentence for clarity and hope it addresses the confusion.<br><br>We have also revised the complete manuscript to address clarity and the issues with punctuation and capitalization.	P 11,<br>Line 297-301

Reviewer: 2
Dr. Lissa M Spencer , Royal Prince Alfred Hospital

Comment	Response	Page, line
Abstract:<br>• Line 29: Spelling mistake 'Objectives'	We have revised the spelling.	-
Introduction<br>• Line 86: define 'Transitional care'. You have not mentioned this in the Abstract. I don't think you should start the Introduction with Transitional care - perhaps you should start with paragraph two.	Thank you for the suggestion. We have defined transitional care and now the introduction section start with paragraph two as you suggested.	P 4<br>Line 86-90
• Line 140: you have stated that 'The aim of the present study was to evaluate the effect of the sPATH intervention on rehospitalization rates and patient activation among patients with heart failure or chronic obstructive pulmonary disease'. However, in the Abstract, you have stated your aims as: 'The aim is to evaluate the effects of a motivational interviewing-based intervention, on rehospitalization and patient activation among patients with congestive heart failure or chronic obstructive pulmonary disease'. The aims should be the same and you	We agree that sPATH needs to be mentioned in the abstract. It has been added and the aim has been revised in the abstract.	P 2<br>Line 29-32<br><br>And the aim at P 5<br>Line 131-133

need to mention sPATH in the Abstract.		
Methods:<br>• Line 153: 'Eligibility criteria were that the patients should have been diagnosed with heart failure or chronic obstructive pulmonary disease…' Rewrite as: 'Eligibility criteria were patients who had been diagnosed with heart failure or chronic obstructive pulmonary disease…'	Thank you for the suggestion on how to rephrase the criteria. We have followed your advice.	P 5<br>Line 143-145
• You need to add how the diagnosis of COPD or HF was made, based on spirometry or echocardiogram results?	Thank you. We agree that this is not clear in the article.<br><br>Unfortunately, our information on patients' diagnoses was retrieved from the medical records, where it was stated what diagnosis the patient had but not which tests were conducted.<br><br>As we agree this is important information, we have added a sentence about this in the method section.	P 5<br>line 148
• Line 162 to 175: This is all Results and needs to go in the Results section.	Thank you for noticing this. We have followed your advice.	P 9<br>Line 258-267
• Line 166: 'a larger block would have balanced less well' This sentence does not really make sense. If you feel this is a limitation – keep limitations for the Discussion section.	We agree that this sentence is confusing, and we have deleted it.	-
• Line 209: You are getting results and methods confused. You need to list the outcomes collected and not comment on the number of Qs completed – this would go in the results.	We agree. The sentence has been moved to the results section.	P 9<br>Line 257-267

• Line 223: Change this heading from 'Baseline Characteristics' to 'Outcomes' and list all outcomes here – make a new paragraph for each outcome.<br><br>Baseline characteristics should not be mentioned until the first paragraph of the Results section.	Thank you. We have changed the heading and removed baseline characteristics to the result section.	P 7<br>Line 204<br><br><br><br>Baseline characteristics in the result section:<br>P 9<br>Line 270-275
• Primary outcome should be listed first, then secondary outcomes and then all the other questionnaires.	Thank you for this comment. We have revised the text based on your suggestion.	P 7<br>Line 205 and 210
Results:<br>• Start the results section with: 'Baseline characteristics for participants in the IG and the CG are reported in Table 1'. Do not write it out – if it is in Tabel 1, that is sufficient. Then go straight to your important between group results.	We thank you for the advice and have revised the section accordingly.	P 9<br>Line 269-275
• Table 1 needs a column showing the between group differences plus 95%CI.	Thank you for stressing this. We have now added 95%CI in the table where it is appropriate, and we have also clarified which analysis was conducted.	P 9, Table 1.
• Line 317: a capital T for Table 2 and Table 3.	We have revised this to capital T.	-
• In the Tables, you could abbreviate intervention group to IG and control group to CG.	Thank you for the suggestion. We now use the abbreviations in Table 2 as this Table benefitted from being clearer. In the other Tables this is not changed in order to enhance for the reader.	P 11<br>Table 2
• When presenting results – make all SD results into whole numbers.	Thank you for the suggestion, we have followed your advice.	P 11<br>Line 297-301<br>P 12<br>Line 314-315
• In Figure 1: what do the authors mean by responders and non-responders?	We understand that the use of "responders" and "non-responders" are confusing. With non-responders we meant that we have no data from these persons. We have revised the phrasing to clarify this misunderstanding.	P 6<br>Line 156-158
• Figure 2: more descriptive to have SD marked on the graph.	Thank you for your suggestion. We chose to display or data in a table as this is common when reporting such data.	P 12

	Thereby Figure 2 is now removed and replaced with Table 4.	
• Do the authors think that more sessions than five in the intervention group, would have improved results would have been different?	We agree that our phrasing seems to suggest this. As this is a hypothetical idea, we have rephrased it in the discussion section to "As it has been reported that more sessions increase behavioral change, future studies could explore if increasing the number of sessions might be helpful for reducing rehospitalization."	P 14<br>Line 372-374
Discussion:<br>• Well done and limitations have answered some of my questions<br>Thank you again for your hard work.	Thank you for this encouraging statement.	-
